# From Biomarkers to Biosensors: Modern Approaches for the Detection of Matrix Metalloproteinases (MMPs)

**DOI:** 10.3390/bios15090603

**Published:** 2025-09-12

**Authors:** Raja Chinnappan, Lohit Ramachandran, Isha Uttam, Marimuthu Citartan, Nidambur Vasudev Ballal, Naresh Kumar Mani

**Affiliations:** 1 College of Medicine, Alfaisal University, Riyadh 11533, Saudi Arabia; rchinnappan@alfaisal.edu; 2 Microfluidics, Sensors and Diagnostics (μSenD) Laboratory, Centre for Microfluidics, Biomarkers, Photoceutics and Sensors (µBioPS), Department of Biotechnology, Manipal Institute of Technology, Manipal Academy of Higher Education, Manipal, Udupi 576104, Karnataka, India; lohit.ramachandran@learner.manipal.edu (L.R.); ishauttam2003@gmail.com (I.U.); 3 Advanced Medical and Dental Institute (AMDI), Universiti Sains Malaysia, Kepala Batas 13200, Penang, Malaysia; citartan@usm.my; 4 Department of Conservative Dentistry and Endodontics, Manipal College of Dental Sciences, Manipal Academy of Higher Education, Manipal, Udupi 576104, Karnataka, India; vasudev.ballal@manipal.edu

**Keywords:** matrix metalloproteinases, biosensors, point-of-care, cancer, periodontitis

## Abstract

Matrix metalloproteinases (MMPs) are a class of extracellular Zn^2+^ peptidases involved in various physiological and pathological processes. These enzymes serve as excellent biomarkers for diagnosing various diseases, including cancer and periodontitis, to name a few. MMP levels also serve as a prognostic marker, which helps determine how much the disease has progressed. However, the current methods used to detect MMPs need a large sample volume, carry a high cost, and are not widely accessible to the public due to these challenges. Biosensing techniques tackle these problems by providing an efficient, cost-effective sensor with great sensitivity. This review provides a comprehensive overview of the latest developments and advancements in detecting MMPs using biosensors that employ various detection mechanisms such as electrochemical, colorimetric, and fluorescence methods. Furthermore, we have discussed the challenges and prospects of using MMPs as diagnostic tools.

## 1. Introduction

The World Health Organization has defined health as a state of complete physical, mental and social well-being and not merely the absence of disease or infirmity [1]. According to the National Institutes of Health (NIH), disease is an abnormal condition that affects the structure or function of part or all of the body and is usually associated with specific signs and symptoms. The origin of diseases can be broadly categorized into these reasons: invasion of microorganisms, induction of autoimmune reaction, induction of allergic reaction, nutrient deficiencies, derangements of metabolic processes, formation, rupture, and thrombotic complication of atherosclerotic plaques, cytogenetic abnormalities, mechanical wear and tear to supportive structures, ingestion of noxious chemicals, and encounter with large kinetic force [2].

Biomarkers, or biological markers, are used to diagnose diseases that can be physiological, chemical, biochemical, or molecular interactions, as stated by WHO [3]. According to the European Medicines Agency (EMA), a biomarker is an objective and quantifiable measure of a physiological process, pathological process, or response to a treatment (excluding measurements of how an individual feels or functions [4]). Biomarkers are used to associate the response with a probability of disease outcome. Besides biological illness, biomarkers can also measure the population’s exposure to toxic chemicals. The cutoff measurement of each biomarker type depends on the disease we are monitoring and highly varies. Biomarkers are used not only to detect diseases but also to determine how much disease has progressed, and they serve as promising tools in treatment. Diagnosis of diseases requires the presence of biomarkers, whose levels can predict the susceptibility of the diseases. These biomarkers vary widely from carbohydrates, proteins, metabolites, and biological compounds. Matrix metalloproteinases (MMPs), also called matrixins, are a family of extracellular Zn^2+^ peptidases that are responsible for the timely breakdown of the extracellular matrix (ECM), which serves essential functions such as embryonic development, morphogenesis, tissue remodeling, and resorption and reproduction [5].

Due to the diverse functions and roles of MMPs, they serve as excellent biomarkers in various pathological processes such as arthritis, cancer, cardiovascular disease, nephritis, neurological disease, breakdown of the blood–brain barrier, periodontal disease, skin ulceration, gastric ulcer, corneal ulceration, liver fibrosis, emphysema, and fibrotic lung disease. They also act as indicators for healthy biological processes, which include embryonic development, blastocyst implantation, organ morphogenesis, nerve growth, ovulation, cervical dilatation, postpartum uterine involution, endometrial cycling, hair follicle cycling, bone remodeling, wound healing, angiogenesis, and apoptosis, to mention of few [6].

By virtue of the various roles of MMPs in many physiological and pathological aspects, they have been adopted in various sensing platforms. Driven by both the functions and their applications in sensors, we aim to provide an overview of the MMPs as the biomarkers and as target molecules in biosensors. Emphasis was given to the major MMPs that were adopted for sensing applications, such as MMP-1, MMP-2, MMP-3, MMP-7, MMP-8, MMP-9, and MMP-14. The significant developments and the state-of-the-art, latest strategies of MMP-based sensors were gleaned to shed more light on the achievements made for the past five years.

### Molecular Mechanism of MMPs

The general catalytic activity of MMPs is driven by a general base mechanism, which involves the activation of a zinc-bound water molecule by the carboxylate group of the conserved glutamate residue in the catalytic pocket. Subsequently, the polarized carbonyl group in the substrate’s scissile bond was attacked by a water molecule [7]. However, due to the discrepancies in substrate specificity, cellular location, and binding, MMPs are a class of multi-faceted biomarkers that serve a multitude of functions; their expression is regulated by many growth factors and cytokines [8]. MMPs are apt for both cancer diagnostics and therapeutics. One role of MMPs is related to Extracellular Matrix (ECM) remodeling, by cleaving and degrading ECM proteins via two main pathways, intracellular, and an extracellular pathway. By virtue of the cleavage and degradation action of ECM proteins by MMPs, their composition, abundance, and architecture are altered [9]. In addition, the remodeling of the ECM by MMPs can also release molecules such as growth factors. Consequently, many physiological processes such as tissue repair and angiogenesis are impacted while also influencing tumor growth, invasion, and metastasis. MMPs are also involved in angiogenesis [10] and regulation of apoptosis in cancer cells [11]. MMP concentrations can vary depending on the specific type of MMPs as well as the type of fluids being analyzed, such as plasma, serum, and saliva [12].

## 2. Types of MMP

**Figure 1:**
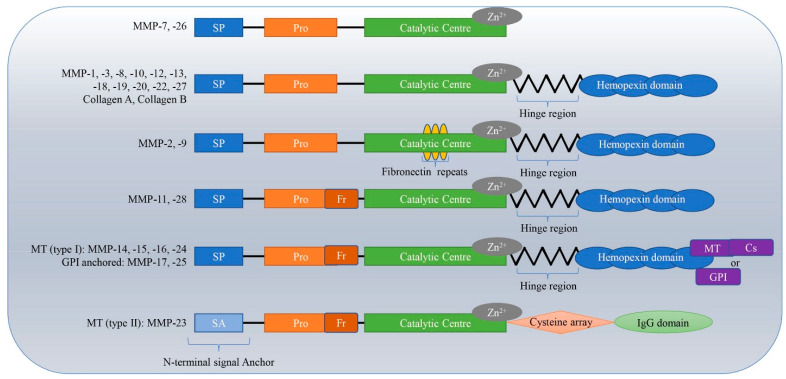
A schematic representation of the different types of MMPs along with their structural architecture (adapted and re-used from [13]).

There are 28 different types of MMPs identified in vertebrates, out of which 23 are human. They are classified into six groups: collagenases, gelatinases, stromelysins, matrilysins, membrane-type MMPs, and other non-classified MMPs [6]. In this section, we will focus on various types of MMPs used as biomarkers for new diagnostic methods for various diseases. Different types of MMPS, along with their domain structures, have been illustrated in Figure 1.

### 2.1. Matrix Metalloproteinases-1 (MMP-1)

MMP-1, or the human fibroblast collagenase, has a major form comprising an unglycosylated form of 57 kDa and a minor glycosylated species of 61 kDa. The gene for MMP-1 is located on the human chromosome 11q22.2–22.3 and is closely connected to eight other MMP genes [14]. From the crystal structure of MMP-1, we observe that the catalytic domain contains a five-stranded beta-sheet, three alpha helices, two zinc ions (one structural and one catalytic), and one calcium [15]. MMP-1 functions as a collagenase-degrading fibrillar collage, which is then transformed into gelatin and further degraded by the other metalloproteinases in the MMP family. Matrix molecules also act as substrates for MMP-1, which include versican, perlecan, aggrecan, casein, nidogen, serpins, and tenascin-C [16]. MMP-1 has demonstrated great significance as a biomarker in disease detection due to the multifunctional role of the molecule. An increase in levels of MMP-1 has correlated to the detection of rheumatoid arthritis due to the increase in collagen degradation [17]. There is also a significant up-regulation of MMP-1 in a wide variety of advanced cancers, and there is also a negative relation between its expression and survival [18].

### 2.2. Matrix Metalloproteinases-2 (MMP-2)

MMP-2, or progelatinase-A, has a molecular weight of 72 kDa [19]. The gene for MMP-2 is located on the human chromosome NC_000016.10 (55478830..55506691) [20]. In the crystallographic structural studies of MMP-2, it was observed that the presence of two chains, A and B, with 165 residues each [21]. Usually secreted as a pro-latent enzyme, MMP-2 functions as a collagenase and degrades the type 4, 5, and 7 collagens and gelatin, fibronectin, and elastin [22,23]. Unlike other MMPs, MMP-2 transcription is not generally induced by TPA or IL-1. Generally, the agents that induce transcription in other MMPs also lack the TATA boxes that are present in most of the other MMP genes [24]. MMP-2 has also expressed great significance in the field of detection of various cancers, such as pancreatic [25], colorectal [26], brain [27], prostate cancer [28], and ovarian cancer [29]. Due to the increased expression of MMP-2 in several human tumors, various studies correlated with this conclusion [30,31,32].

### 2.3. Matrix Metalloproteinases-3 (MMP-3)

MMP-3, or stromelysin-1, with a molecular weight of 57 kDa, plays a variety of functions, making it a very useful biomarker for detecting a broad spectrum of diseases. MMP-3 consists of around 475–478 amino acids in mammals, and its gene sequence is highly conserved across various species [33]. MMP-3 plays a role in cellular fibrinolytic activity [34] and neurodegeneration due to increased cell stress [35] is also involved in multiple biological processes, such as cell differentiation and inflammation, since it is a stromal shaping agent, besides its general function in digesting the extracellular matrix [36]. MMP-3 also has a plethora of functions, such as acting as a common inflammatory factor that promotes inflammation, and it plays a complicated role in the nervous system, a few of which are peripheral nerve injuries directly related to the oversecretion of MMP-3 [37] MMP-3 gene polymorphism is also found to have a direct relation with increased occurrence and development of cardiovascular diseases such as rheumatic heart disease and atherosclerosis [38,39].

### 2.4. Matrix Metalloproteinases-7 (MMP-7)

MMP-7, or matrilysin-1, with a molecular weight of 29.667 kDa, consists of a 267 amino acid sequence length. Unlike the other members of its MMP family, it lacks a hinge region and hemopexin-like domain [40]. The mucosal and exocrine glands produce MMP 7 and epithelial cells, and these regulate alpha-defensin activity in the innate defense mechanism [41]. MMP-7 also serves as a biomarker for various diseases, such as pancreatic cancer. Khulmann et al. have demonstrated an overexpression of MMP-7 in pancreatic cancer [42], Liu et al. investigated the expression of MMP-7 in non-small cell lung cancer, and the results showed a significant correlation between increased expression of MMP-7 and tumor proliferation. It is also observed to be a prognostic factor [43]. Research done by Maurel et al. has concluded that MMP-7 acts as an independent prognostic factor in advanced colorectal cancer, and it can be used as a biomarker for detecting colorectal cancer [44].

### 2.5. Matrix Metalloproteinases-8 (MMP-8)

MMP-8, collagenase-2, or neutrophil collagenase, with a molecular weight of 42 kDa and an amino acid sequence of 467, is mainly produced in neutrophils. Even though MMP-8 is a collagenase, it has a substrate specificity different from its counterpart, MMP-1 [45]. MMP-8 is involved in the digestion of aggrecan, gelatins, and cartilage link proteins, which are part of the ECM. MMP-8 is also responsible for the digestion of non-ECM substrates such as α_2_-macroglobulin, α_1_-antichymotrypsin, α_1_-antiproteinase inhibitor, insulin-like growth factor binding protein (IGF-BP)-2 and IGF-BP-3, connective tissue growth factor (CTGF), and pro-TGF-β [36]. MMP-8 is involved in the digestion of aggrecan, gelatins, and cartilage link proteins, which are part of the ECM. MMP-8 is also responsible for the digestion of non-ECM substrates such as α_2_-macroglobulin, α_1_-antichymotrypsin, α_1_-antiproteinase inhibitor, insulin-like growth factor binding protein (IGF-BP)-2 and IGF-BP-3, connective tissue growth factor (CTGF), and pro-TGF-β [36]. MMP-8 also serves as a biomarker for various diseases, and salivary MMP-8 is used as a biomarker in detecting periodontitis [46]. Elevated MMP-8 levels in serum samples of patients are used as an indicator of gastric cancer and used in the diagnosis of the same [47]. MMP-8 also serves as an essential diagnostic tool used in the detection of colorectal cancer, and it is also associated with poor prognosis of the same [48].

### 2.6. Matrix Metalloproteinases-9 (MMP-9)

MMP-9, or gelatinase-B, with a molecular weight of 92 kDa, is synthesized from its gene present in chromosome 20q13.12., with 13 exons and 12 introns and an amino acid sequence of 730 [49]. MMP-9 is involved in various biological functions such as the proteolytic degradation of the ECM, cleaving cell surface proteins, altering cell-cell and cell–ECM interactions, and cleaving proteins in the extracellular environment. It also plays an essential role in basement membrane degradation [50,51,52,53,54]. MMP-9 also serves as a biomarker for various diseases, such as in the detection of breast cancer, where it is found to be present in the urine of breast cancer patients [55,56]. The research done by Tian et al. also established the finding that MMP-9 is present in pancreatic juice and serves as a biomarker for pancreatic cancer [57]. MMP-9 is present in elevated levels of serum of lung cancer, although not in the bronchial lavage fluid of patients, and serves as a biomarker for lung cancer [58,59]. MMP-9 is an important prognostic indicator for bladder cancer and can be a vital biomarker tool. MMP-9 is an important prognostic indicator for bladder cancer and can be a vital biomarker tool [60]. MMP-9 overexpression is also found to directly correlate with the presence of T3-T4 node-negative colorectal cancer [61]. Pro MMP-9 activity is found to have a significant relationship with advanced ovarian cancer and is used as a biomarker and is also used to predict the survival rate [62]. Increased expression of MMP-9 in urine samples is found to help detect prostate cancer and serves as an essential biomarker for early detection [63]. Overexpression of MMP-9 in human glioma tumors is used as a biomarker in detecting brain cancer [64]. MMP-9 is also an essential biomarker in other diseases, such as inflammatory bowel disease and dry eye disease [65,66]. Thus, we can observe that, unlike the other metalloproteinases, MMP-9 plays a significant role as a biomarker in almost all types of cancers and various other diseases.

### 2.7. Matrix Metalloproteinases-14 (MMP-14)

MMP-14, or membrane-type MMP with a molecular weight of 66 kDa, is synthesized from its gene in chromosome 14q11.2, with ten exons. Unlike other secreted matrix metalloproteinases, this membrane protein is expressed at the cell surface. MMP-14 is also responsible for activating MMP-2 [67]. MMP-14 also helps in the cleaving of gelatin, fibronectin, and laminin. MMP-14 is also an internalized protein; clathrinated-mediated endocytosis [68] and caveolae-mediated endocytosis [69] help regulate this. MP-14 also serves as a biomarker for various diseases, such as ovarian cancer [69]. MMP-14 is also an important prognostic tool for digestive system carcinoma [70].

## 3. Detection of MMPs Using Various Biosensing Platforms

**Figure 2:**
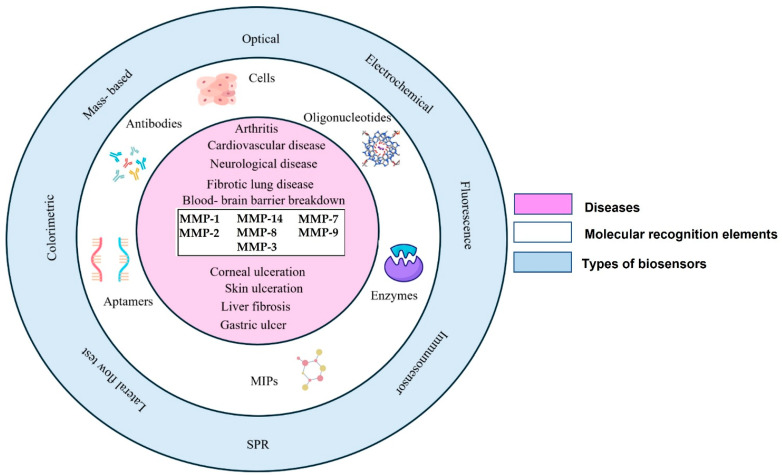
Schematic diagram of various biosensing methodologies used for the detection of MMPs (MMP-1, MMP-2, MMP-3, MMP-7, MMP-8, MMP-9, MMP-14) demonstrated for the diagnosis of multiple diseases using different molecular recognition elements. Various molecular recognition elements have been deployed for the diagnostics of MMPs, with various formats of detection ranging from fluorescence, electrochemical, optical, mass-based, colorimetric, lateral flow assay, SPR to immunosensing-based approaches.

Biosensors are integrated receptor-transducer devices that analyze a biological response by converting it into an electric signal. They must be specific and independent of various physical parameters like pH, temperature, etc. In a biosensor, biological elements like enzymes, antibodies, or aptamers are integrated with an electronic component that detects, records, and transmits the data in the given environment. A typical biosensor consists of an analyte, a bioreceptor, a transducer, internal electronics (amplifier and processor for signal processing), and a display (a PC or a printer). Biosensors have been applied in many fields, starting from the food industry, to detect artificial sweeteners using electrochemical techniques, to the medical field, where fluorescent biosensors play a vital role in drug discovery and cancer. Figure 2 illustrates the development of various types of biosensors using different kinds of recognition elements integrated with multiple transducers using MMPs as biomarkers for the diagnosis of cardiovascular diseases, arthritis, neurological diseases, fibrotic lung diseases, blood-brain barrier breakdown, and several other diseases.

### 3.1. Biosensors for Matrix Metalloproteinases-2 (MMP-2)

**Figure 3:**
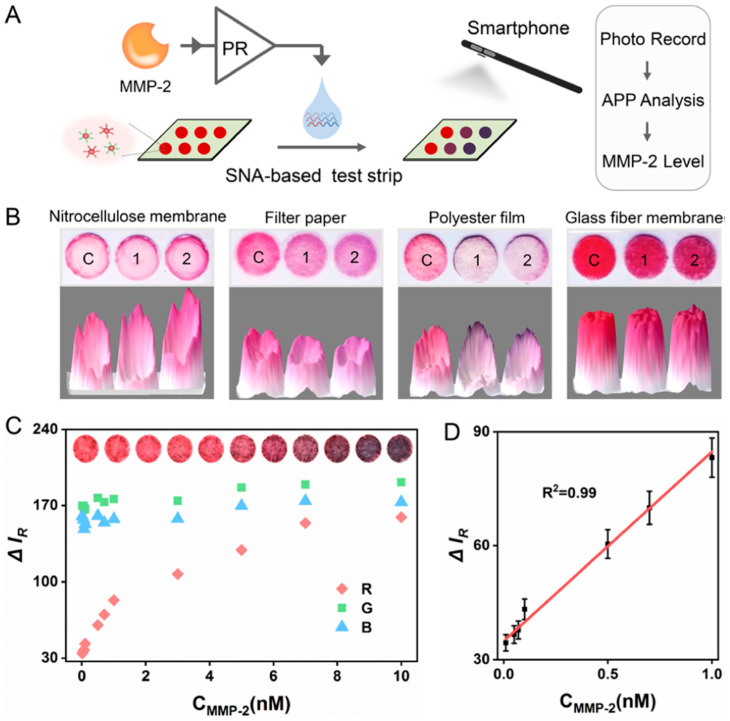
Schematic of a smartphone-based colorimetric detection system for the analysis of Target Protease Biomarkers. (**A**) A schematic representation of a colorimetric system based on paper that uses data processing and a smartphone. (**B**) A shift in the paper’s color: C stands for control, 1 for 0.5 U T7 RNAP transcription products, and 2 for 1.0 U T7 RNAP transcription products. (**C**) Colorimetric signal fluctuation at various MMP-2 concentrations. The original photos are displayed at the top. (**D**) Colorimetric response signal calibration plot versus MMP-2 concentration [71].

Liu et al. developed a novel colorimetric protease assay by integrating proteolysis-responsive transcription with spherical nucleic acids (SNAs) (Figure 3) [71]. Peptide, which is a substrate of MMP-2 was used as a linker between T7 RNA polymerase and its inhibitor protein (catalytically inactivated T7 lysozyme). In the presence of the target MMP-2, the linker was cleaved by the target protein, which released the enzyme, T7 RNA polymerase, restoring its activity. As a result, multiple RNA molecules were produced, causing the aggregation of gold nanoparticle (AuNP)-based spherical nucleic acids (SNAs), shifting the absorption peak from 525 to 620 nm. The solution changed its color from red to blue. In the absence of the target MMP-2, the T7 RNA polymerase remains inactive, causing no production of RNA molecules. Consequently, there was no shift in the absorption peak of the AuNP-SNAs, as they were stable and separated from each other, rendering the solution to remain red in color. Their method demonstrated exceptional sensitivity in detecting MMP-2, achieving a remarkable limit of detection (LOD) of 0.2 ng/mL.

Nawaz et al. used a multiple signal sensing probe based on a highly luminous nanosized Cu-PTC metal–organic framework (MOF) to construct an electrochemical sensor for detecting MMP-2 in blood and cell samples [72]. The shape, crystallinity, and elemental composition of the MOF were evaluated. The fluorescence intensity is reduced as a result of the electrostatic interaction between the negatively charged Cu-PTC MOF probe and the positively charged fluorescence MMP-2 substrate. The substrate cleaves when MMP-2 is present, resulting in a turn-on and fluorescence. The sensor exhibits a linear detection range of 1 to 175 ng/mL and a limit of detection of 0.8 ng/mL.

**Figure 4:**
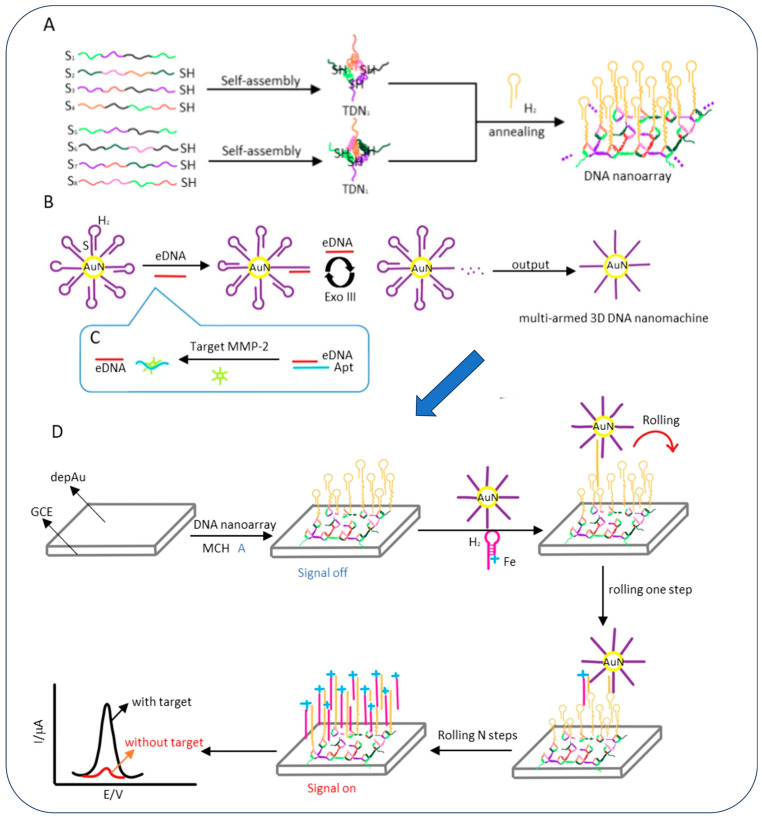
(**A**) Using two different tetrahedral DNA structures as tracks, the DNA nanoarray is assembled. (**B**) Using Exo III-assisted cDNA recycling amplification to create the multi-armed 3D DNA nanomachine. (**C**) Target MMP-2 is transformed into cDNA. (**D**) Target MMP-2 detection using an electrochemical biosensor [72].

Yao et al. developed an electrochemical sensor for the detection of MMP-2 by using a giant-sized DNA nanoarray assembled from two kinds of tetrahedral DNA structures as the DNA track (Figure 4) [72]. This contraption served as a track for the multi-armed three-dimensional (3D) DNA nanomachine, thus enabling efficient and fast signal transduction and amplification. Unlike conventional DNA walkers that walk on random DNA tracks, the meticulously fabricated multi-armed 3D DNA nanomachine exhibited faster reaction speed and better walking efficiency due to the rigidity and orderliness of the tetrahedral DNA nanoarray structure. This sensor was capable of ultrasensitive detection of the MMP-2 biomarker with an LOD of 11.4 fg/mL and a detection time of 60 min, thus making it suitable for point-of-care applications.

A fluorescence-based detection of MMP-2, a tumor marker, is challenging to detect at low abundance in the early stages using traditional methods [73]. The sensor utilizes aggregation-induced emission (AIE) and incorporates a two-step MP/NPs-SLIPS sensing system designed for ultrasensitive detection of MMP-2. In this system, the aggregation of AIE residual is enhanced through electrostatic absorption by negatively charged nanoparticles (NPs) and the confined space formed by the self-assembly of NPs to photonic crystals (PCs) on slippery lubricant-infused porous substrates (SLIPS). This unique approach allows for the detection of MMP-2 with an LOD of 3.7 ng/mL and a detection range of 1.6 μg/mL to 50 ng/mL.

Using a cell membrane-anchored ratiometric upconversion nanoprobe (UCNPs-Cy3/Pep-QSY7/Ab) for in situ imaging of MMP-2 secretion, Fang et al. created a novel fluorescent sensor for MMP-2 detection [74]. The anti-EGFR functionalization of the nanoprobe enables it to identify tumor cells with precision and react rapidly to MMP-2 in the local secretory region. Cy3 luminescence at 580 nm is recovered as a result of the MMP-responsive cleavage of Pep-QSY7. For MMP-2 detection, this luminescence is then compared to an internal standard of UCNP emission at 654 nm. This sensor demonstrated encouraging outcomes for both in vivo imaging of metastatic lymph nodes and in situ monitoring of MMP-2 secretion from MDA-MB-231 and MCF-7 cells. The sensor’s detection range is 1 to 100 ng/mL, with a limit of detection of 0.51 ng/mL. Using methionine (Met)/N-acetyl-L-cysteine (NAC) templated copper nanoclusters (Met/NAC-Cu NCs) with a tunable near-infrared region (NIR) electrochemiluminescence (ECL) emission, a highly sensitive electrochemiluminescence sensor has been developed for the detection of MMP-2 [75].

In order to obtain Cu NCs, NAC served as both a template and a reductant of cupric. NAC was connected to the surface defect regulator Met via an -S-S- bond. By adjusting the molar ratio of Met to NAC, this link increased the surface defect of Cu NCs and made it possible to continuously control the maximum ECL emission. As a result, Cu NCs with an ECL emission between 680 and 750 nm were produced. Additionally, by placing hairpins evenly and neatly to boost local concentration, they developed a fast target-triggered catalytic hairpin assembly (CHA) recycling amplification technique. The reaction rate and signal amplification efficiency were greatly increased by this tactic. The LOD of the sensor was 1.65 fg/mL.

Jaric et al. developed an immunosensor for the detection of MMP-2 [76]. The research focused on the optimization of electrochemical reduction in the production of thin-film-modified gold electrodes; it was also observed that 20 cycles of cyclic voltammetry were optimal in attaining superior activation of graphene oxide into electrochemically reduced graphene oxide (ERGO). This sensor was used in the detection of MMP-2; the sensor utilizes anti-MMP-2 aptamers as the biorecognition element. The sensor demonstrates an excellent limit of detection (LOD) of 3.32 pg/mL and a versatile range of 10 pg/mL to 10 ng/mL.

An excellent point-of-care (POC) diagnostic sensor was developed for detecting MMP-2 [77]. The POC system consisted of light excitation and its photocurrent measurement, which was achieved using a miniature integrated circuit board. Graphitic carbon nitride (g-C_3_N_4_) and gold nanoparticles loaded on indium tin oxide (ITO) electrodes were employed as photoactive materials, and signal amplification elements were used as elements of the photoelectrochemical sensor. The gold nanoparticle immobilized the MMP-2-specific cleavage peptide, modified with bovine serum albumin (BSA) at the terminal end. In the presence of MMP-2, the peptide was specifically hydrolyzed and cleaved. This resulted in parts of the peptide chain and BSA being detached from the electrode, leading to a decrease in steric hindrance and an increase in the photoelectrochemical current; a linear trend is established between the photochemical current and the logarithm of MMP-2 concentration. The sensor exhibits an LOD of 0.48 pg/mL and a range of detection of 1 pg/mL to 100 ng/mL.

Zhang et al. developed a biosensor for MMP-2 detection and proposed its use in anti-aging research [78]. They presented a novel method for MMP-2 detection using a bipedal DNA walker. Peptide nucleic acid is used as the substrate for MMP-2, releasing a bandage strand with two DNA strands acting as legs and, thus, a bipedal walker. The bipedal walker “leg” initiates the bipedal walker isothermal amplification strategy, which involves entropy-driven DNA walking and catalytic hairpin assembly on a PEI@Ru(bpy)_3_^2+^-Ti_3_C_2_@AuNPs-modified electrode. The movement of the walker’s leg produces an electrochemiluminescence signal, which is measured by the sensor and helps quantify the MMP-2 present. The sensor has an impressive LOD of 4.2 pg/mL.

### 3.2. Biosensors for Matrix Metalloproteinases-3 (MMP-3)

Guo et al. developed a new magnetoelastic biosensor for the detection of MMP-3 used as a biomarker for osteoarthritis [79]. A novel flexible ME (magnetostrictive/electrostrictive) was used as a substrate instead of the traditional rigid substrates already in use. The magnetostrictive material TbDyFe is mixed with a paraffin solution to create a TbDyFe fluid, which is then reacted at high temperature with SEBS (styrene-ethylene-butylene-styrene) in a specific ratio to fabricate the film. The film acts as the substrate to immobilize the MMP-3 antibodies. The binding of MMP-3 to its antibodies generates stress across it, leading to significant changes in its magnetic permeability and impedance. To enhance the sensitivity of the biosensor, the doping concentration of TbDyFe is optimized; it is observed that the sensor demonstrated 0.76 ng/mL of LOD and a range of detection of 0.76–10,000 ng/mL.

**Figure 5:**
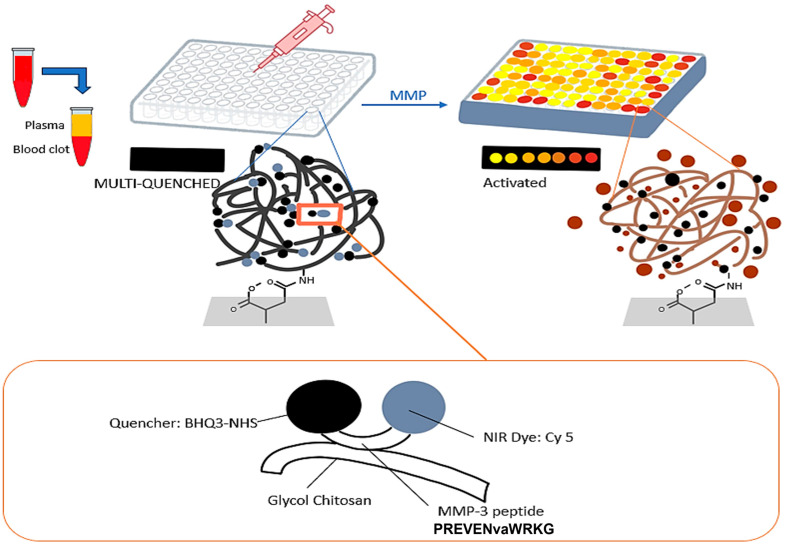
Scheme for detecting active MMP-3 in RA patient peripheral blood. The kit uses an MMP-3 specific substrate conjugated with Cy5 and BHQ-3, immobilized on a glycol chitosan polymer in a 96-well plate. Initially, the probe is quenched. Upon adding active MMP-3, fluorescence is restored, indicating the concentration of active MMP-3 [81].

An environmentally friendly sensor was constructed for the detection of MMP-3 [80]. The sensor is based on the principle of hydrogen evolution reaction (HER). The sensor works by utilizing the overpotential of the Ag-Cu bimetallic HER catalyst as the signal, eliminating the need for further redox reactions. The sensor exhibits an excellent limit of detection of 2.02 fg/mL and a wide linear range of detection from 0.001 to 100 ng/mL. Lee et al. developed an assay for detecting rheumatoid arthritis using MMP-3 as the biomarker (Figure 5) [81]. They use an MMP-3-specific protease-activated probe immobilized in an in vitro kit and also used in cell staining for flow cytometry analysis methods to improve the accuracy of clinical diagnosis. Plasma from 269 rheumatoid arthritis (RA) patients, 49 osteoarthritis patients, and 30 healthy volunteers was used to validate the sensor. The sensor demonstrated an excellent LOD of 114 ng/mL.

Guo et al. constructed another magnetoelastic biosensor for detecting MMP-3, which acts as a biomarker for osteoarthritis [79]; the sensor is based on a metal glass alloy 2826MB used to measure the MMP-3. The sensor works by measuring the frequency change due to the magnetostrictive effects. The device consists of an ME chip immobilized with an MMP-3 antibody and an electromagnetic coil. The sensor is susceptible to mass change; thus, it can detect the frequency shifts caused by mass change due to the binding of MMP-3 to its antibody. The biosensor has already been successfully used in detecting MMP-3 from joint fluid of OA patients, with a significant LOD of 30.7 ng/mL and a range of detection of 30.7 to 2000 ng/mL.

### 3.3. Biosensors for Matrix Metalloproteinases-7 (MMP-7)

Chen et al. developed a sensor against MMP-7 consisting of a peptide-modified MnFe_2_O_4_ ratiometric fluorescent nanoprobe for noninvasively visualizing MMP-7 both in vitro and in vivo [82]. The nanoprobe uses a fluorescein isothiocyanate (FITC)-modified peptide containing the specific motif VPLSLTMG for MMP-7 cleavage, which is conjugated with MnFe_2_O_4_ nanoparticles (NPs) to produce a Förster resonance energy transfer (FRET) system for sensing MMP-7. Rhodamine B (RhB)-modified targeting peptide is also immobilized on the nanoparticle surface, which serves as a reference for forming a ratiometric fluorescence system with the FITC dye. In the presence of MMP-7, FITC detaches from the MnFe_2_O_4_ surface, producing FITC fluorescence, which is measured. The sensor exhibits an excellent LOD of 2 ng/mL and 436 cells and a range of detection of 2 ng/mL to 300 ng/mL in buffer and 5 × 10^2^ to 1 × 10^4^ cells.

**Figure 6:**
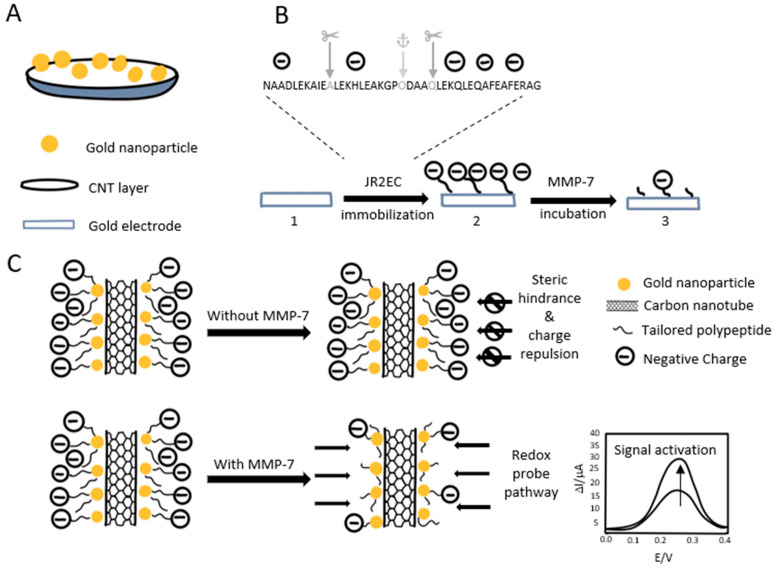
(**A**) The sensor construction is shown schematically. (**B**) Procedures for building the biosensor: (1) Gold electrode prior to immobilization of polypeptides, (2) following immobilization of polypeptides, and (3) following reaction with MMP-7. (**C**) Principle of operation: By blocking the redox probe, the immobilized polypeptide suppresses the electrochemical signal. The resulting cleavage of the peptide by the MMP-7 enzyme results in signal recovery [83].

Palomar et al. developed a new innovative method to detect MMP-7 by using a peptide-decorated gold nanoparticle/carbon nanotube electrochemical sensor (Figure 6) [83]. The sensor can operate in diluted human urine or serum, making the sensor noninvasive; the sensor exhibits high specificity and stability compared to conventional electrodes, and the fabrication is also simple and cost-effective. The sensor exhibits an LOD of 6 pg/mL and has a range of 1 × 10^−2^ to 1 × 10^3^ ng/mL

To accurately detect MMP-7, Margardia et al. created a novel and inventive detection technique that uses hydrogel-based technology. Molecularly imprinted polymers (MIPs), which were previously constructed around red-emitting quantum dots, are combined with blue-emitting carbon dots (CDs) to create a dual emissive fluorescence probe. Molecularly imprinted polymers (MIPs) that were previously constructed around red-emitting quantum dots (QDs) were combined with blue-emitting carbon dots (CDs) to create a dual-emissive fluorescent probe [84]. The MIPs are the biorecognition elements conjugated to cadmium telluride QDs, which act as the sensing platform. The red quantum dots reduced fluorescence intensity while the analyte concentration increased, while the blue-emitting carbon dots maintained a constant fluorescent intensity, which serves as the internal control. Under a 365 nm UV lamp, the color changes from red to blue due to it being a function of MMP-7 concentration. The MIP@QDs are then incorporated into a cellulose hydrogel containing CDs, which serve as reference probes, forming imprinted ratiometric hydrogels (imprinted rHGs). This innovative sensor exhibits an excellent limit of detection of 4.11 × 10^−12^ g/mL and a wide linear range of detection between 1.49 × 10^−11^ and 1.92 × 10^−9^ g/mL in 1000-fold diluted human serum.

Li et al. developed a sensitive protease sensor capable of detecting multiple metalloproteinases, MMP-7 and MMP-2 [85]. The sensor contains two DNA-peptide conjugates, which include specific protease cleavage sites and trigger DNA, and also two reporter DNAs, which are tagged with a fluorophore (Cy3 or Cy5) and a quencher (BHQ2). After encountering the specific MMP, the cleavage reaction occurs, leading to a cascade reaction that finally ends with the release of the fluorophores, which is easily measured using total internal reflection fluorescence-based single molecule detection. This sensor has excellent sensitivity due to its proteolytic cleavage activity; it exhibits an LOD of 34.2 pg/mL for MMP-7 and 66.6 pg/mL for MMP-2.

**Figure 7:**
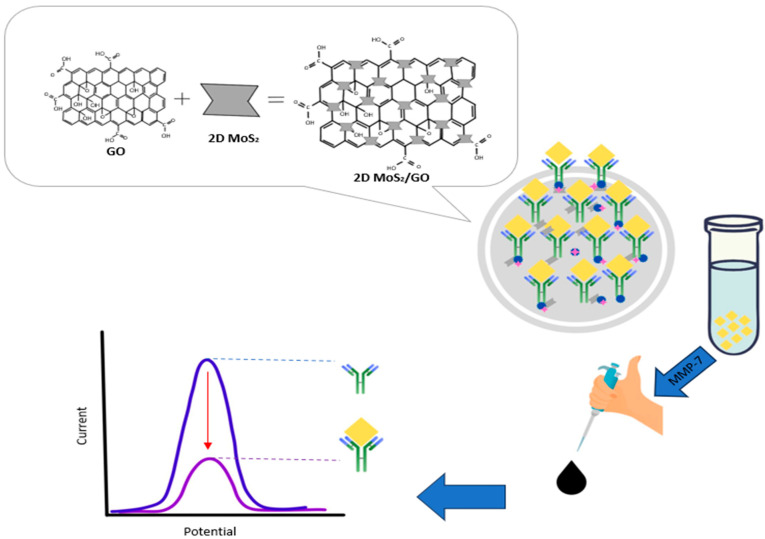
Schematic representation of an immunosensor for detecting MMP-7 using methylene blue (MB), 2D MoS_2_, and graphene oxide [87].

An ultrasensitive antifouling biosensor fabricated for the quantitative detection of MMP-7 [86]. The sensor contains a multifunctional peptide combined with urease@zeolite imidazole frameworks (urease@ZIFs), which results in an antifouling electrode interface. This was then used in conjunction with a sodium alginate-graphene oxide-Pb^2+^ (SA-GO-Pb^2+^) gel. This system was combined with a novel carboxyl-rich pyrrole-doped ZIF (ZIF-Py) loaded with urease. In the presence of urease@ZIF-Py, CO_2_ was produced, which reacted with Pb^2+^ present in the gel to form PbCO_3_ precipitation, which led to a noticeable decrease in the conductivity of the sensing interface. This was then measured for the presence of MMP-7. The sensor exhibited an ultrasensitive LOD of 24.34 fg/mL and a wide linear range of detection of 0.1 pg/mL to 100 ng/mL. Yaiwong et al. developed an immunosensor for the detection of MMP-7 (Figure 7) [87]. The sensor involved the adsorption of methylene blue onto a two-dimensional molybdenum disulfide (2D MoS_2_)/graphene oxide (GO) nanocomposite coated on a screen-printed carbon electrode (SPCE). The nanocomposite has unique qualities such as a large surface area, good electrical conductivity, and rapid electron transfer, which increase the sensor’s performance. Anti-MMP-7 capture antibodies are immobilized onto the MB/2D MoS_2_/GO nanocomposite surface through electrostatic interaction, which enables the detection of MMP-7. The sensor exhibits an LOD of 0.007 ng/mL and a linear range of detection from 0.010 to 75 ng/mL.

### 3.4. Biosensors for Matrix Metalloproteinases-8 (MMP-8)

Guido et al. developed a surface plasmon resonance (SPR) based on polymer optical fibers (POF) for the detection of MMP-8 [88]. The SPR-POF biosensor was immobilized with a specific antibody, which helped create the surface-assembled monolayer for capturing MMP-8. A spectrophotometer was used to quantify the amount of MMP-8 present, samples taken from saliva were successfully validated, and the biosensor demonstrated an LOD of 9.9 ng/mL and a linear range of detection from 22.9 ng/mL to 489.9 ng/mL. He et al. developed a disk-shaped lateral flow immunoassay for detecting MMP-8 from gingival cervical fluid (GCF), a biomarker for periodontitis [89]. The sensor utilizes green core–shell upconversion nanoparticles (G-UCNPs). As a luminescent probe for the detection of MMP-8. The sensor exhibits an LOD of 5.455 ng/mL and has its use case in point-of-care diagnostics due to its cheap and quick diagnosis.

Öztürk et al. developed a new point-of-care diagnostic dipstick method for the analysis of active MMP-8 (aMMP-8) [90]. The sensor works on the principle that proteins from the GCF get eluted into the buffer and move along the dipstick, which then encounters blue antibody-labelled particles and bind to them. If there is a sufficient quantity of aMMP-8 present, a blue line is visible on the stick within 5 min, making it very quick and helpful in the diagnosis of periodontitis. Two blue lines indicate a positive test, while one negative line means it is negative. The test’s cutoff point is 20 ng/μL, and the test has a sensitivity of 83.9% and a specificity of 79.2%.

**Figure 8:**
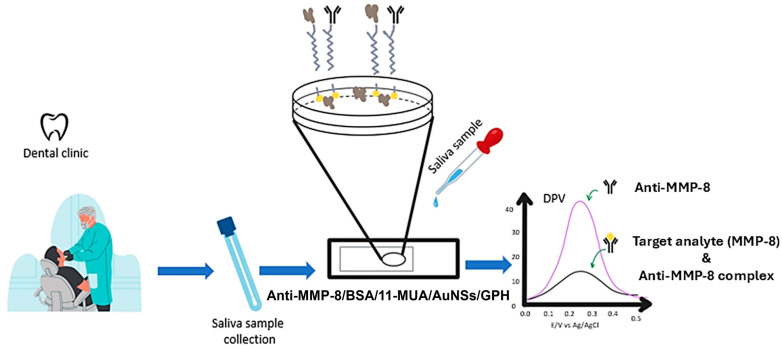
Schematic representation of a disposable voltametric immunosensor for detecting MMP-8 to diagnose periodontitis [91]. The sensor is based on a graphene (GPH) screen-printed electrode (SPE) functionalized with gold nanospheres (AuNSs) and antibodies against the MMP-8 protein (anti-MMP-8). The formation of the MMP-8 protein-antibody complex impedes the electron transfer of the redox probe, causing the decrease in the DPV signal. However, in the absence of the target, the unhindered electron transfer of the redox probe causes the DPV signal to increase.

Tortolini et al. developed a novel voltametric immunosensor for the detection of MMP-8 (Figure 8) [91]. The sensor employs a graphene screen-printed electrode (SPE), which is then coated with gold nanospheres (AuNSs) and antibodies against MMP-8 (anti-MMP-8). Thus, when the sensor encounters MMP-8, it binds to the anti-MMP-8, which causes a disturbance and is measured. The sensor demonstrates an LOD of 1.0 ± 0.1 ng/mL and a linear range of detection of 2.5–300 ng/mL.

Annuziata et al. developed an MMP-8 sensor [92] similar to the sensor developed by Guido et al. [88] It employs the same surface plasmon resonance principle, antibody self-assembled monolayers created on modified plastic optical fibers, which helps in the detection, unlike the sensor made by Guido et al. This sensor is capable of measuring MMP-8 and MIP-1α. A spectrophotometer is connected to the sensor for the quantification. Johannsen et al. developed a detection method using magnetic beads and fluorescent beads for capture and detection, respectively, to detect MMP-8 [93]. This method has several advantages, such as skipping the washing step and measuring fluorescence in the bound-free phase, allowing for quantitative detection. The novel method boasts a limit of detection of 0.24 ng/mL and a range of detection of 0.47–30 ng/mL.

### 3.5. Biosensors for Matrix Metalloproteinases-9 (MMP-9)

A lateral flow test for the detection of MMP-9 has been demonstrated by Kim et al. [94] The LFT was made with a plastic backing, sample pad, nitrocellulose (NC) membrane, and an absorbent pad. This LFT is used to detect periodontitis. Thus, the adsorbent pad was specifically designed for the collection of oral fluids, which were then transported to the sample pad where they interacted with the conjugate pad. The conjugate pad was sprayed with Au-NP-conjugated anti-MMP-9 antibodies that interact with the salivary MMP-9. Thus, they migrate to the NC membrane, where the results are read; the diagnostic ability of the test was 0.82 (with a sensitivity of 0.92% and specificity of 0.72%).

**Figure 9:**
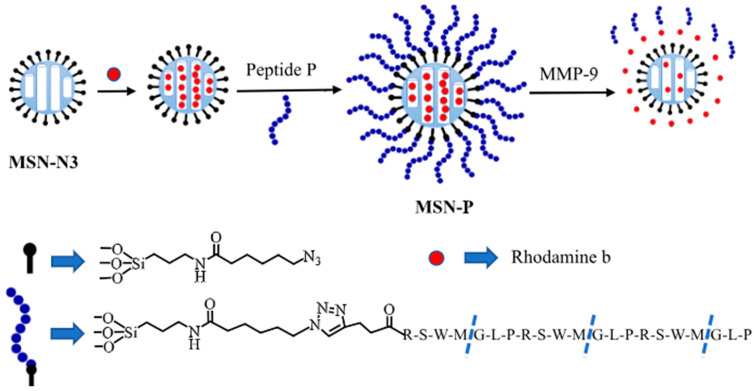
Experimental design of MMP-9 substrate capped MSN. The external surface of MSN was modified by the azide group. Rhodamine b was loaded and capped using the peptide substrate. The introduction of MMP-9 releases the dye by cleaving the substrate [95].

Liu et al. developed an assay for the detection of MMP-9 used in the detection of inflammatory bowel disease [95]. The assay utilizes a cap and release method, which uses mesoporous silica nanoparticles (MSNs) to detect fecal MMP-9 and serum TNF-α. MMP-9 peptide substrates act as caps as they attach to dye-loaded MSNs (Figure 9). In the presence of MMP-9, the substrate cleaved and the dye was released. The free dye in the solution was detected by a fluorometer; the assay exhibits an LOD of 1.1 μg/mL.

**Figure 10:**
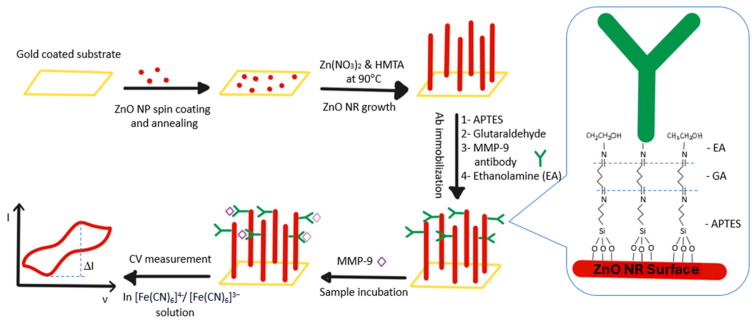
Schematic diagram illustrating the working of a ZnO nanoparticle/nanorod-based label-free electrochemical immunosensor for rapid detection of the MMP-9 biomarker. The formation of the MMP-9-antibody complex decreases the current peak due to impedance of the electron transfer while in the absence of the target there is no blockade to the electron transfer, causing the current peak to increase [96].

Shabani et al. developed an electrochemical immunoassay for the detection of MMP-9 biomarkers in serum (Figure 10) [96]; unlike the other methods, they measured the mass charge transfer resistance of electrodes using cyclic voltammetry and electrochemical impedance spectroscopy. Antibodies were immobilized on zinc oxide nanoparticles and ZnO nanorod electrodes, which makes the assay possible. The assay has an LOD of 32.5 μA/(decade × cm^2^) and a detection range of 1–1000 ng/mL.

Lu et al. developed a novel silicon nanowire-based biosensor for detecting MMP-9 in human tears to diagnose dry eye disease [66]. The biosensor was based on silicon nanowire-based field-effect transistor (SiNW FET) devices, which help quantitatively analyze MMP-9. The research also established a high correlation between this method and the industry-tested enzyme-linked immunosorbent assay standard, with a diagnostic sensitivity of 86.96% and specificity of 90%. Ghosh et al. developed an ultra-sensitive chemiresistive sensor for the detection of potentially blinding eye diseases using MMP-9 as the biomarker using actual tear samples [97]; the device uses synthesized vanadium disulfide nanowires and MMP-9 antigens to help in the detection. Ghosh et al. also identified various factors contributing to baseline drifts of the chemiresistive sensor, including nanowire coverage on the interdigitated microelectrode. The sensor response duration and the effect of MMP-9 protein in different matrix solutions led to a significantly low limit of detection (LOD) of 0.2746 fg/mL and a wide range of detection of 10 fg/mL to 1 μg/mL.

Perumal et al. developed a sensor for the detection of MMP-9 [98], which is used to track the wound healing process; they developed a biofunctionalized, low-cost, and scalable plasmonic SERS substrate using cellulose fiber, which was used to detect MMP-9 using immunoassay method. The sensor has a wide detection range of 10–5000. Rainu et al. developed a sensor for detecting MMP-9 using dual-sensitive nanoprobes [99]. Carbon nanoparticles coated with MMP-9 peptide sequences were used to make the nanoprobes. Carbon nanoparticles were chosen for their intrinsic fluorescence properties, and the probes also had a peptide sequence that was susceptible to cleavage by MMP-9. When the peptide sequence gets cleaved by the MMP-9, the detached molecule produces a fluorescence signal, which indicates the presence of MMP-9. Intensive testing was done in vitro via these nanoprobes to distinguish tumor-like microenvironments from the non-cancerous ones. Rainu et al. suggest that this device could be used as a non-invasive imaging tool for real-time visualization of tumor margins.

Arevalo et al. developed an electrochemical sensor for the detection of MMP-9, which is used in breast cancer detection from cancer cell lysates and serum samples [100]. The sensor uses magnetic microbeads (MBs)-based sandwich immunoassay for amperometric determination of MMP-9 at screen-printed carbon electrodes (SPCEs). A capture antibody is immobilized on the microbeads to capture the antigen that formed a sandwich with a detector antibody. The sandwich complex was conjugated with commercial streptavidin-horseradish peroxidase (Strep-HRP) polymer, which helps in detection; the sensor exhibits a limit of detection (LOD) of 2.4 pg/mL.

Kim et al. developed an aptamer-based sensor for the detection of MMP-9 [101]. The sensor cleverly utilizes a DNA aptamer that specifically targets MMP-9 in a tumor micro-environment and has a high affinity and sensitivity. Gold nanospheres, plasmonic in nature, are modified with the MMP-9 aptamers and its complementary sequences, which enables the nanosphere to bind to MMP-9 through DNA displacement and hybridization. This leads to a plasmon coupling effect, which can be identified with ultrasound-guided photoacoustic (US/PA) imaging, which facilitates the detection of MMP-9. In vitro testing was done to demonstrate the efficiency of the biosensor.

### 3.6. Biosensors for Matrix Metalloproteinases-14 (MMP-14)

Duan et al. developed a sensor for the detection of MMP-14 using MDA-MB-231 cancer cells as the sample and the electrogenerated chemiluminescence (ECL) method [102].

An inhibitory peptide (AP1) was used as the capture probe, while another ruthenium complex-tagged inhibitory peptide served as the signal probe (Ru-AP2). The sensor was developed by attaching the Cys group of AP1 to a functional fullerene-chitosan (C60-Chit) nanocomposite-modified glassy carbon electrode through covalent bonding. Upon the binding of MMP-14, the signal probe binds to the MMP-14, creating a sandwich structure, which leads to the generation of the ECL signal. The sensor demonstrated an LOD of 0.008 pg/mL and a range of 0.05–7 pg/mL.

## 4. Conclusions and Future Perspectives

**Figure 11:**
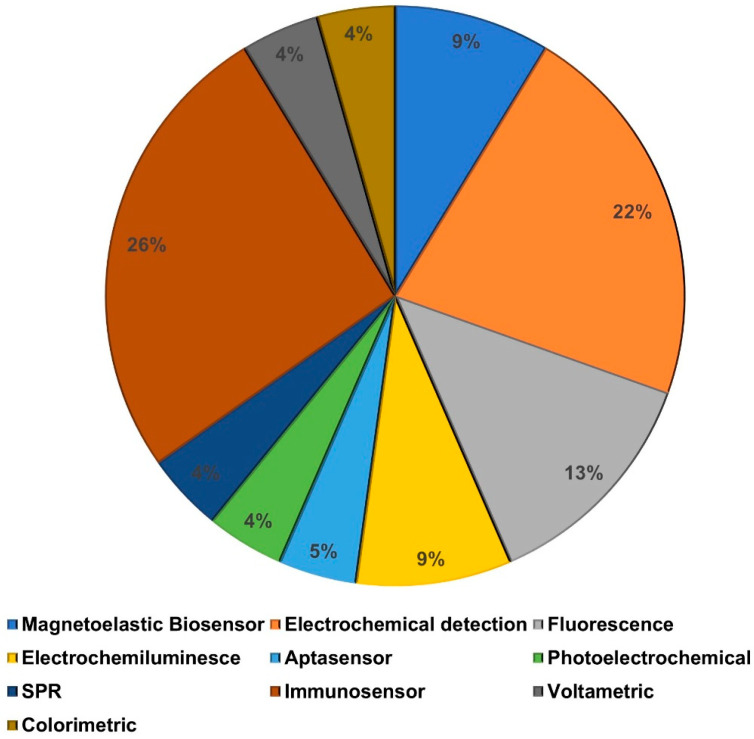
The breakdown of the sensors developed for the detection of MMPs.

Biomarkers are vital in the diagnosis and treatment of various ailments. In this review, we have studied one such biomarker, matrix metalloproteinases, a family of extracellular Zn^2+^ peptidases responsible for the timely breakdown of the extracellular matrix (ECM). This review covers the different types of MMPs used as biomarkers and the latest advancements in the detection mechanism of MMP within the past five years. There has been significant research conducted on this topic leading to the fabrication of sensors with significantly lower limits of detection, up to the femtogram per milliliter level, and a greater range of detection, which led to the creation of ultrasensitive sensors for the detection of MMP (Table 1). The high accurate sensors can be used in the early diagnosis of diseases, thus helping in the treatment and improving the rate of recovery. The most frequently used sensors are immunosensors, owing to the availability of antibodies against various MMPs for their specific detection (Figure 11). From this review, it is evident that the performance of each biosensor differs due to the discrepancies in the type of MREs used, the binding affinity of these MREs against their target molecules, the immobilization efficiency of these MREs onto the surface of the diagnostic platform, type of signal read-out used, and type of body fluids used for the diagnostics of MMPs.

While biosensors show immense promise in the detection of MMPs, one primary challenge to address is the lack of actual companies implementing these sensors, which limits their accessibility in clinical settings. To overcome this problem, collaboration between the researchers and the industry should be strengthened to facilitate these sensors in the commercial market, and more effort should be put into making these sensors more point of care (POC) in nature. POC testing will facilitate easier detection and rapid output in either bedside or outpatient settings; developing more portable, user-friendly devices that are easy to use will increase the real-life use of these sensors. POC diagnostics refer to the decentralized nature of diagnostics, whereby diagnostics are no longer confined to hospitals but can be performed anywhere the end users intend to [104].

The inherent limitations or challenges of assays that thwart their applications in POC settings can be identified by referring to the ASSURED guidelines, which stands for affordable, sensitive, specific, user-friendly, rapid and robust, equipment-free, and deliverable. One of the most challenging issues is related to the stability of the molecular recognition elements (MREs) as bioreceptors. MREs that are prone to degradation due to temperature or enzymatic actions would render the POC devices non-functional. Therefore, the selection of stable molecular recognition elements such as aptamers would enhance the durability and robustness of POC diagnostics. In the context of diagnostics related to MMPs, aptamers specific against these biomarkers can be integrated into POC devices. Conventional assays for cancer diagnostics, such as histochemistry, are primarily tedious and expensive and require trained personnel, impeding their transition to POC diagnostics. Venturing into label-free and equipment-free assays, such as gold nanoparticles and lateral flow assays, can revolutionize cancer diagnostics based on MMPs, engendering POC diagnostic assays that are able to fulfill the ASSURED criteria. Colorimetric assays, apart from being independent of any equipment, can also be cost-saving and facile, able to be carried out even by the patient themselves.

Integration of these biosensors with microfluidics and imaging methods can further improve their performance and their applicability in real-world settings. This integration can help in the real-time monitoring of MMP, which can be used to monitor processes like wound healing, thus making it beneficial in chronic wound management. Biosensor research in multiplex detection capable of simultaneous detection of multiple MMP markers can help open up new research in diagnosis and treatment monitoring. Despite a lot of research work having been reported, there is no commercially available biosensing device for POC testing in the market. Bench-to-bed commercialization of this work in the form of a miniaturized device would advance the field of diagnosis of various cancers and may achieve breakthroughs in disease management in the future.

Even though there have been several recent developments in biosensors for MMP detection, new multidisciplinary techniques are opening the door to better diagnostics. For instance, programmable, multiplexed detection is made possible by DNA origami-based logic gates, which allow for flexible responses depending on the MMP combinations [105]. Similarly, CRISPR-based biosensors are perfect for point-of-care MMP diagnostics, particularly in infectious diseases and cancer, because they provide single-molecule sensitivity with extremely high specificity [106]. Real-time, non-invasive monitoring of MMP activity in saliva, tears, and sweat could be made possible by integrated wearable biosensors. By categorizing patients according to biomarker profiles and real-time physiological feedback, the combination of these biosensors with AI and machine learning could contribute to improving diagnostic accuracy. Together, these developments indicate a future where medicine is increasingly precise and individualized.

## 5. Challenges

One of the key challenges in studying biofluids is their heterogeneity, regardless of whether they are invasive or non-invasive, which makes it difficult to control or predict biomarker concentrations across individuals or conditions. A practical approach to address this issue is to first conduct a preliminary validation study to identify the biofluid that exhibits the most consistent and standardized biomarker levels and then use that as the preferred medium for downstream analysis. For instance, in the context of this review, saliva has shown relatively stable levels of MMP-8 and MMP-9, making it a promising and reliable biofluid for MMP-based diagnostic studies [107]. For the improved performance of the sensor, biological fluid such as saliva is diluted to mitigate the non-specific interaction between the non-target molecules and the MREs due to the reduction of their concentration. In parallel to this, the act of diluting retains the specific interaction between the target MMP-8/MMP-9 with the MREs due to their much higher binding affinity as compared to the binding strength of the non-specific interactions. As a result, the high performance of MMP-8- and MMP-9-based sensing is ensured. Another significant challenge is the development of new point-of-care (POC) devices that do not contribute further to the growing medical waste problem. Most current POC and diagnostic devices are manufactured from single-use plastics, primarily due to the need for safe disposal of contaminated materials and their low production cost; for instance, a standard operating room produces waste equivalent to a family of four producing in a week. All this makes the healthcare sector the second largest contributor to waste collected in landfills after the food industry [108]. To address this issue, future innovations must prioritize the use of sustainable materials, including textile-based or paper-based diagnostic platforms, which offer biodegradability and reduced environmental burden without compromising diagnostic performance. Another challenge is adapting biosensors for multiplex detection of MMPs. In the case of MMP-2 and MMP-9, both these isoforms have similar catalytic action related to gelatinase activity but differ in terms of gene regulation. MMP-2 is produced by a wide range of cell types like macrophages and endothelial cells, whereas consecutive production of MMP-9 is restricted to neutrophils and eosinophils in adults. However, the literature confirms that MMP-2 and MMP-9 are often co-released into the extracellular matrix during inflammation, fibrosis, and cancer progression, supporting the need for multiplex biosensing. Given that MMP-2 and MMP-9 are regulated differently and induced by different cytokines, only one isoform may dominate under some conditions. Thus, for multiplex detection, prior studies have to be made regarding target MMPs. In the case of only isoform, detection must be solely based on the particular isoform of MMP. However, in the event of several active isoforms of MMPs, simultaneous detection of these MMPs can be achieved by using MREs specific to each of the isoforms. These MREs can then be integrated into methods such as sandwich ELISA, paper-based microfluidic sensors, or a matching aptamer cocktail-based assay [109].

## Figures and Tables

**Table 1:** Various bioanalytical methods used for the detection of different MMPs.

Biomarkers	Materials Used for Sensing Application	Method of Detection	LOD	Detection Range	Advantages	Disadvantages	References
MMP-2	Gold nanoparticle (AuNP)-based spherical nucleic acids (SNAs)	Colorimetric	3.3 pM	0.01–10 nM	Portable, making it suitable for POC settings High LOD, sensitivity and specificity	Sample preparation requirements: Requires specific buffer conditions and incubation steps, which might limit field use without proper tools. Prone to gold nanoparticles aggregation in the presence of any contamination in the sample	[71]
	Cu- pyridine-2,4,6-tricarboxylate (PTC) metal–organic framework (MOF)	Electrochemical Detection	0.8 ng/mL	1 ng/mL to 175 ng/mL	Dual mode detection: Both fluorescence and electrochemical sensing, thereby improving readability and flexibility. Large surface area: Facilitating substrate interaction and signal interaction.	Visual, naked-eye detection is not possible due to complex instrumentation, reducing accessibility for low-resource settings. Limitation in portability and ease-of-use. Instrument for signal read-out can be expensive	[103]
	Multi-armed three-dimensional (3D) DNA nanomachine	Electrochemical Detection	11.4 fg/mL	-	High structural rigidity and order The multi-armed structure creates a high local concentration of recognition elements, which enhances target recognition efficiency and boosts electrochemical signal efficiency	Instrumentation required reducing portability	[72]
	Intensified aggregation-induced emission (AIE) by slippery lubricant-infused porous substrates (SLIPS)	Fluorescence	3.7 ng/mL	1.6 μg/mL–50 ng/mL	Potential for high-throughput	Fluorescence signal prone to photobleaching	[73]
	Cell membrane-anchored radiometric up conversion nanoprobe (UCNPs-Cy3/Pep-QSY7/Ab)	Fluorescence	0.51 ng/mL	1 ng/mL to 100 ng/mL	Provides a universal platform for the study of proteases and contributes for tumor assessment	Potential damage to cells due to exposure to short-wavelength (high energy) excitation light	[74]
	Methionine (Met)/N-acetyl-L-cysteine (NAC) templated copper nanoclusters (Met/NAC-Cu NCs)	Electrochemiluminescence	1.65 fg/mL	-	Regulation of optical behavior of metal nanocluster based ECL emitters Less light damage, penetrates deeper into tissue Strong potential for highly sensitive biosensing and clear ECL imaging.	Limited structural clarity due to smaller size of copper nanoclusters.	[75]
	Electrochemically reduced graphene oxide (ERGO) thin film-modified gold electrodes	Aptasensor	3.32 pg/mL	10 pg/mL–10 ng/mL	Highly specific Reusable	Potential non-uniform film formation due to drop-casting deposition. Dielectric nature of deposited GO limits electrochemical performance.	[76]
	Graphitic carbon nitride/gold nanoparticles loaded on indium tin oxide electrodes	Photoelectrochemical	0.48 pg/mL	1 pg/mL to 100 ng/mL	The system is portable and suitable for POC applications. Comparable performance to Laboratory Equipment.	Limited excitation performance with blue and green LEDs; system works best only with purple (400 nm) meaning limited compatibility with other photoactive materials that don’t absorb well at 400 nm.	[77]
	PEI@Ru(bpy)_3_^2+^-Ti_3_C_2_ @AuNPs-modified electrode	Electrochemiluminescence	80.6 fM	-	Highly sensitive and specific	Potential high cost associated with modified electrodes	[78]
MMP-3	TbDyFe/polystyrene-poly (ethylene-butylene)-polystyrene block copolymer (SEBS) film	Magnetoelastic Biosensor	0.76 ng/mL	0.76 ng/mL to 1000 ng/mL	Wide range of detection and highly sensitive	Higher doping led to an adverse effect of mechanical	[79]
	Ag-Cu bimetallic hydrogen evolution reaction (HER) catalyst	Electrochemical detection	2.02 fg/mL	0.001 ng/mL to 100 ng/mL	Exceptional consistency and dependable performance.	Requires precise optimization of multiple parameters	[79]
	Phorbol 12-myristate 13-acetate (PMA)-activated plasma	Fluorescence	1.9 nM	0.07–30 nM	Ease of use and highly sensitive	Cross reactivity and low natural levels	[81]
	Magnetoelastic (ME) chip immobilized with MMP-3 antibody and an electromagnetic coil	Magnetoelastic	30.7 ng/mL	30.7 ng/mL to 2000 ng/mL	Wide linear range and verified consistent performance.	Possible magnetic field interference due to reliance on magnetostrictive materials	[79]
MMP-7	MnFe_2_O_4_ nanoparticles (NPs) modified fluorescein isothiocyanate (FITC) labelled MMP-7 substrate	Fluorescence	0.1 nM	0.1 nM to 15 nM	Low sustained toxicity	Fluorescence signal prone to photobleaching	[82]
	Nanocomposite of carbon nanotubes (CNTs) and electrogenerated gold nanoparticles (GNPs) electrode	Electrochemical detection	6 pg/mL	0.01 ng/mL to 1000 ng/mL	User-friendly and cost-effective	Limited long-term stability	[83]
	Imprinted ratiometric hydrogels: (generated from blue emitting carbon dots (CDs), molecularly imprinted polymers (MIPs) and assembled around red emitting quantum dots (QDs)	Fluorescence	4.11 pg/mL	14.9 pg/mL to 1.92 ng/mL	Increased selectivity and higher sensitivity	Limited data in real human serum. Limited storage stability data, with performance evaluated only up to 20 days.	[84]
	Fluorophore-quencher labelled DNA-peptide conjugates with specific protease cleavage sites	Fluorescence	1.71 pM		Simultaneous monitoring of several MMP targets in complex samples	Complexity of method	[85]
	Multifunctional peptide with urease@zeolite imidazole frameworks (urease@ZIFs) using sodium alginate-graphene oxide-Pb^2+^ (SA-GO-Pb^2+^) gel	Electrochemical detection	24.32 fg/mL	0.1 pg/mL to 100 ng/mL	Excellent stability and successful clinical application	Poor conductivity of biocomponents	[86]
	2D MoS2/GO nanocomposite deposited screen-printed carbon electrode (SPCE)	Immunosensor	0.007 ng/mL	0.010 ng/mL to 75 ng/mL	High specificity and stability	Preparation of materials is complex involving complex surface chemistry	[87]
MMP-8	Anti-MMP-y antibody functionalized surface plasmon resonance (SPR) plastic optical fiber (POF)	SPR	9.9 ng/mL	22.9 ng/mL to 489.9 ng/mL	Rapid response time Immediate on-site analysis	Clinical validation needed	[88]
	Disk-like lateral flow immunoassay strip (LFIS) using green core-shell upconversion nanoparticles (G-UCNPs) as luminescent probe	Immunosensor	5.455 ng/mL	-	Multiplexing capability and simplified operating procedure	Larger sample size is needed	[89]
	DipStick/Antibody	Immunosensor	83.9%(specificity)	-	High diagnostic accuracy with rapid results Good agreement with standard lab methods	Cross-sectional study design No grading information used	[90]
	Anti-MMP-8 functionalized graphene (GPH) screen-printed electrode (SPE) functionalized by gold-nanospheres (AuNSs	Voltametric immunosensor	1 ng/mL	2.5 ng/mL to 300 ng/mL	High selectivity, High reproducibility High stability	More studies with randomized designs are needed Larger sample sizes are required Current understanding of MMP-8 is limited Further research is required to confirm prognostic use	[91]
		Immunosensor	0.24 ng/mL	0.47 ng/mL to 30 ng/mL	Fast detection High sensitivity	Reduced signal for MMP-8 due to biological interactions Potential interference from biomolecular interactions	[93]
MMP-9	Capturing magnetic beads and fluorescent detection beads agents	Immunosensor	0.38 ng/mL	0.47 ng/mLto 30 ng/mL	Adaptable to various biomarkers High reproducibility	Reduced signal for MMP-8 and MMP-9 in triplex due to biological interactions	[93]

## Data Availability

Data sharing is not applicable to this article as no new data were created or analyzed in this study.
